# Translating theory into practice – dealing with pre- and post- menopausal women patients with schizophrenia

**DOI:** 10.1192/j.eurpsy.2021.1596

**Published:** 2021-08-13

**Authors:** N. Semenova, O. Karpenko, B. Kazakovtsev, V. Krasnov

**Affiliations:** 1 Psychiatry, Moscow Research Institute of Psychiatry – a branch of V.Serbsky National Medical Research Centre for Psychiatry and Narcology, Moscow, Russia, Moscow, Russian Federation; 2 First Episode Department, Psychiatric Hospital № 1 named after N.A. Alekseev, Moscow, Russian Federation; 3 Department Of Epidemiological And Organizational Problems Of Psychiatry, The Serbsky National Research Center for Psychiatry and Narcology, Moscow, Russian Federation; 4 Psychiatry, Pirogov Russian National Research Medical University, Moscow, Russian Federation

**Keywords:** Psychoeducation, women, Menopause, schizophrénia

## Abstract

**Introduction:**

Much has been written about psychosocial treatments (psychoeducation) in schizophrenia. However, for the psychiatric hospital as an organization wishing to create a service guided by an international wave of research there is a need for solutions which are practical and effective in addressing the gender issues and women patients’ needs.

**Objectives:**

This paper looks at and describes the process employed to develop a guidance document to enable the psychoeducation to provide information and to offer support to its women patients (pre- and post- menopausal age) in dealing with schizophrenia. Essential to this project was the understanding that the guidance would be easy to understand and practical whilst maintaining its strong foundation of research and good practice.

**Methods:**

Women patient profile in a schizophrenia group hospitalized at the Moscow-based Psychiatric Hospital was analyzed.

**Results:**

Females of pre- and post- menopausal age actually outnumber males. Such women patients are unique in their needs and demands for health services. This is important subgroup, and some psychosocial interventions should be developed for them. Mental health professionals should be familiar with the unique health problems of these women, and the potential that psychoeducation have to increase their health awareness (information on menopause and aging, oestrogens and depression, on other somatic and psychological influences around menopause, on effects of physical activity etc.).

**Conclusions:**

The implications of this guidance document to enable the psychoeducation for mental health promotion are discussed, in particular the gendered nature of perception of psychosocial treatments in schizophrenia.

